# Supplementary material for: Targeting IFNα to tumor by anti-PD-L1 creates feedforward antitumor responses to overcome checkpoint blockade resistance
Source: Nat Commun. 2018 Nov 2;9:4586. doi: 10.1038/s41467-018-06890-y (PMC6214895; doi:10.1038/s41467-018-06890-y)
Supplement: Supplementary file 1 — Supplementary Information [file 41467_2018_6890_MOESM1_ESM.pdf]

Targeting IFNα to tumor by anti-PD-L1 creates feedforward antitumor responses to overcome checkpoint blockade resistance

Liang et al.

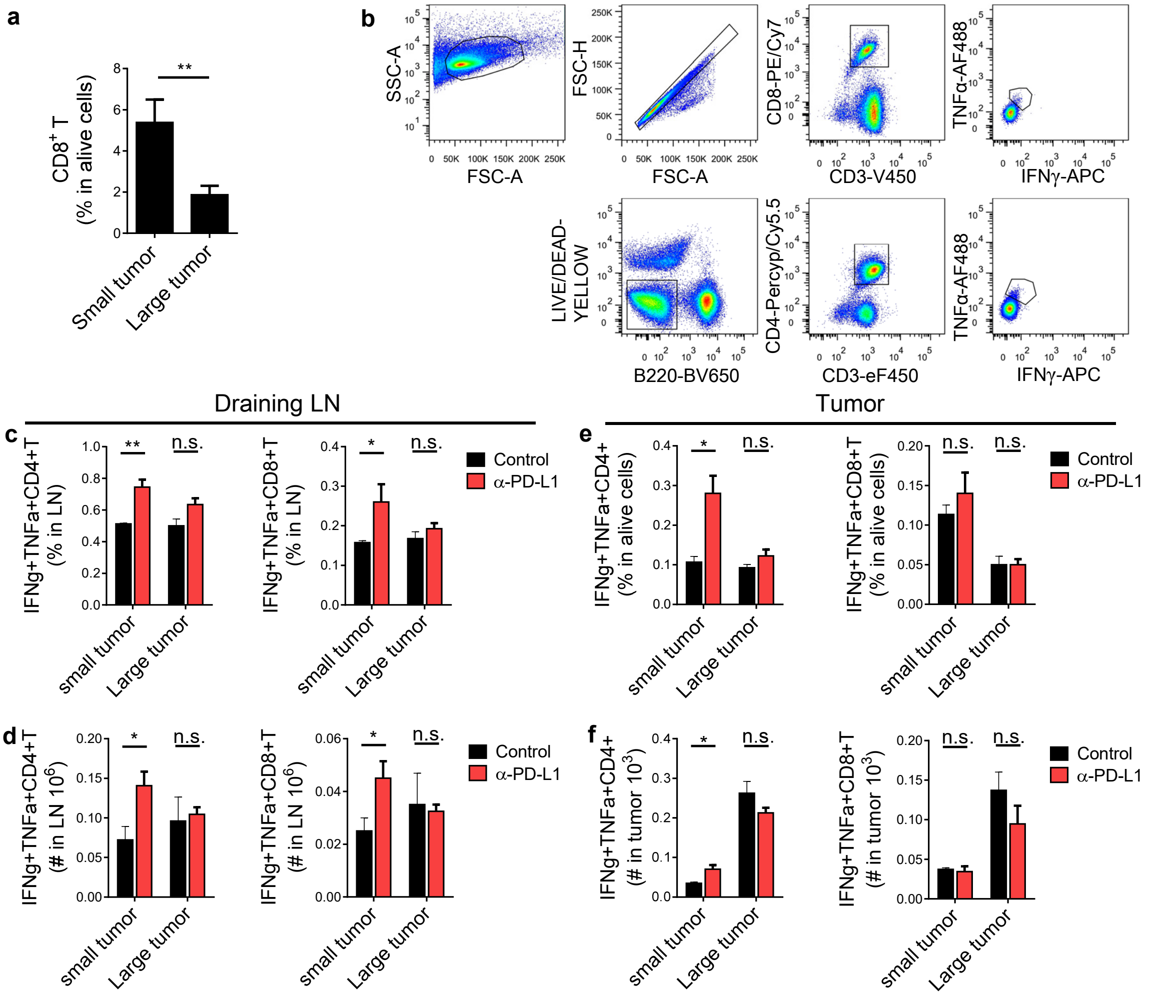

Supplementary Figure 1: PD-L1 blockade activate the effector function of T cells in small tumor but not advanced tumor. (a) Tumor tissues were collected from Balb/c mice bearing early stage tumors (<50 mm^3) or advanced A20 tumors (>100 mm^3). The frequency of CD3$^+$CD8$^+$ T cells within alive tumor cells were determined by FACS. (b) Gating strategy for IFNγ$^+$TNFα$^+$CD4$^+$ and CD8$^+$ T cells from the tumor-draining lymph node. (c-f) Balb/c mice (n=4) bearing early stage tumors or advanced A20 tumors were treated intraperitoneally (i.p.) with 200 µg of anti-PD-L1. After 3 days, mice were i.p. injected with 500 µl of 0.5 mg/ml Brefeldin A solution. 6 hours later, draining lymph node and tumor tissues were collected and digested. FACS staining was performed. (c) The frequency of IFNγ$^+$TNFα$^+$CD4$^+$ or CD8$^+$ T cells within alive dLN cells. (d) The absolute number of IFNγ$^+$TNFα$^+$CD4$^+$ or CD8$^+$ T cells within dLN. (d) The frequency of IFNγ$^+$TNFα$^+$CD4$^+$ or CD8$^+$ T cells within alive tumor cells. (f) The absolute number of IFNγ$^+$TNFα$^+$CD4$^+$or CD8$^+$ T cells within per mg tumor tissues. %, the frequency of cell subsets. #, the absolute number of cells subsets. Data indicate mean ± SEM and are representatives of two independent experiments. *, $p<0.05$; **, $p<0.01$; ***, $p<0.001$.

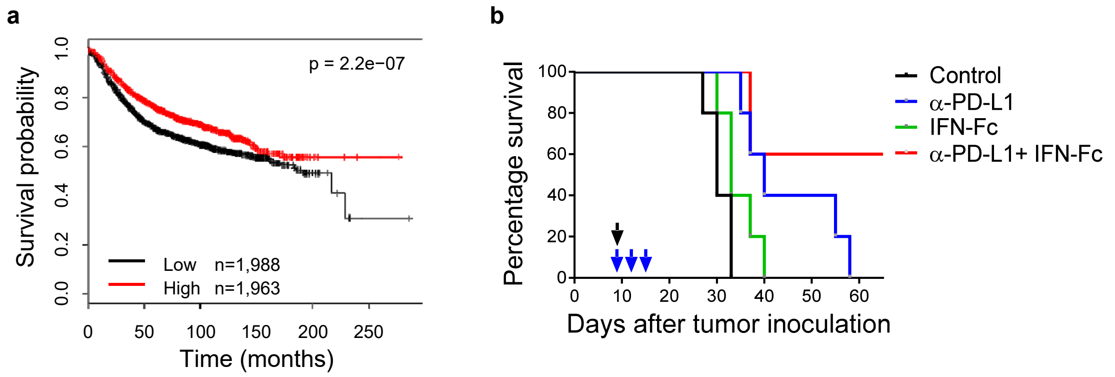

Supplementary Figure 2: IFNA1 expression level positively correlates with better survival in human breast cancer patients. (a) Kaplan-Meier curve was generated by comparing patients with high (n=1,963) vs low (n=1,988) IFNA1 expression levels. Statistical significance was determined by the log-rank test. (b) Survival curves of MC38 tumor bearing mice in **Fig.1e**.

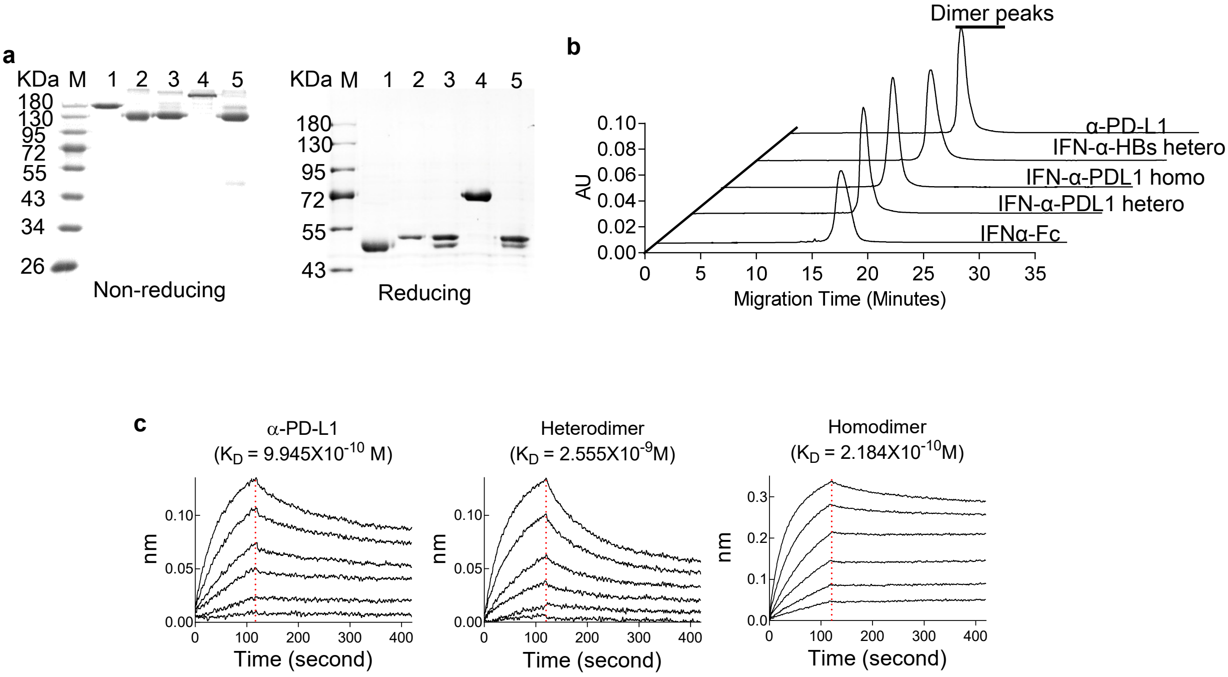

Supplementary Figure 3: Biochemical characterization of the fusion protein. (a) Fusion proteins were expressed in 293F cells and were analyzed by non-reducing (left) and reducing (right) SDS-PAGE after purification. M, molecular weight marker; 1, α-PDL1-Fc; 2, IFNα-Fc; 3, IFNa-anti-PD-L1 heterodimer; 4, homodimer; 5, IFN-α-HBs. (b) CE electropherograms of indicated proteins. (c). Binding curves of α-PDL1-Fc, IFN-α-PDL1 heterodimer or IFN-α-PDL1 homodimer to the immobilized PD-L1-Fc-biotin by BLI. The constants were determined by dynamic-analysis model. Data is a representative of two independent experiments.

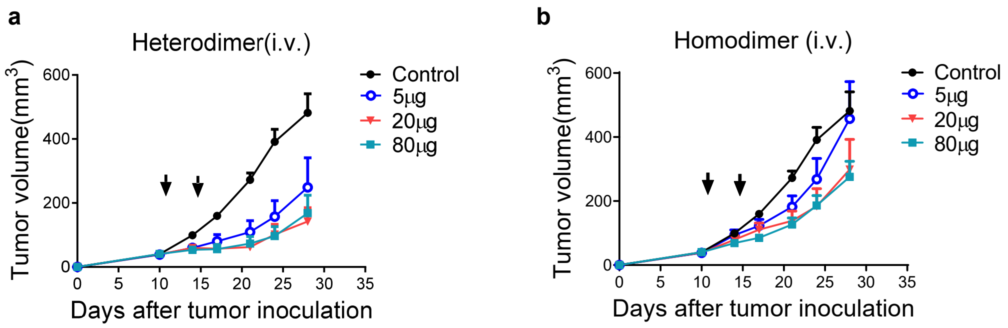

Supplementary Figure 4: Titration of fusion proteins for antitumor effects. Balb/c mice (n=4 to 6) were inoculated with 3×10^6 A20 cells. After tumor established, mice were treated with indicated doses of IFN-α-PDL1 heterodimer or homodimer protein by i.v. injection twice (day 11 and 15). Tumor size was measured twice per week. Data is a representative of two independent experiments.

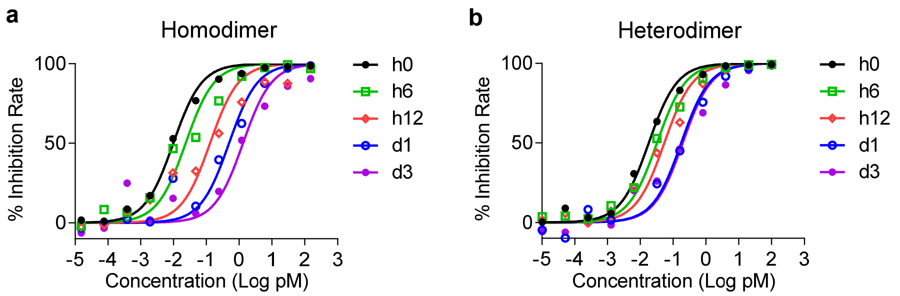

Supplementary Figure 5: The stability of IFN-α-PDL1 fusion protein. To compare the stability, IFN-α-PDL1 homodimer (a) or heterodimer (b) were added into mouse serum and incubate at 37℃ for indicated time. The activity of proteins was detected by IFN anti-viral infection bioassay.

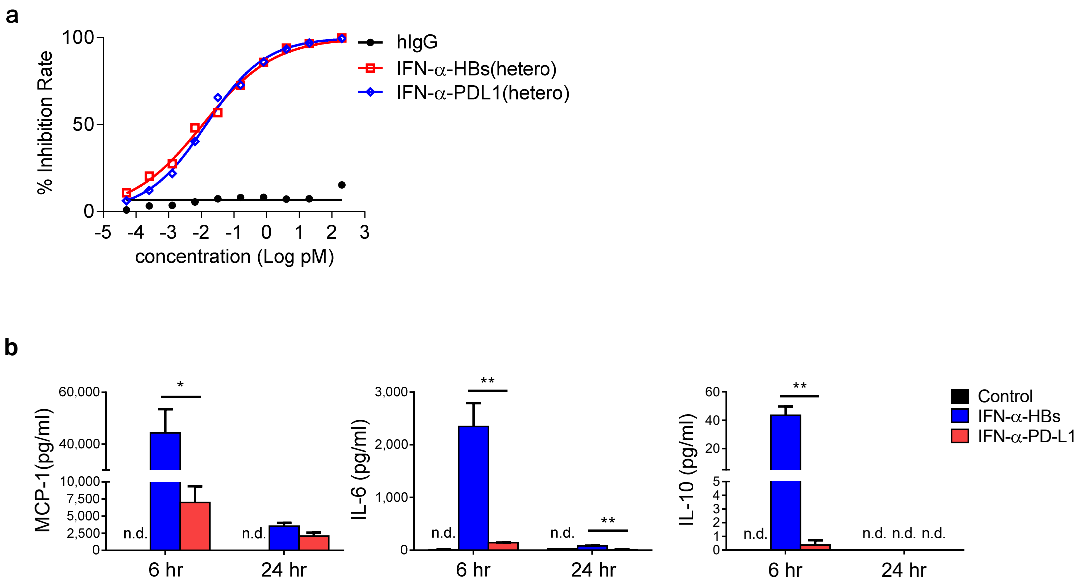

Supplementary Figure 6: IFNα-anti-PD-L1 heterodimer specifically targets tumor tissues and induce less toxicity. (a) The bioactivity of IFNα-α-PD-L1 and control IFNα-α-HBs protein was measured by an antiviral infection biological assay. (b) MC38 tumor-bearing mice were treated as in Figure 3c. Serum was collected at 6 and 24 hours after injection. Cytokine levels in the serum were measured by CBA. Data indicate the mean ± SEM and is a representative of two experimental repeats.

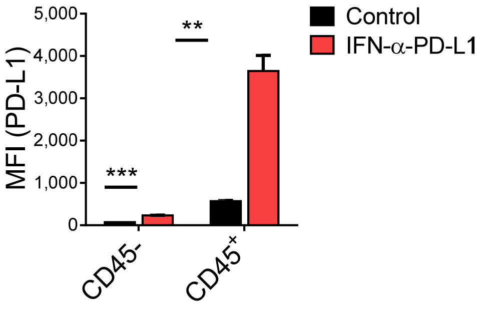

Supplementary Figure 7: IFNα-anti-PD-L1 upregulates PD-L1 within tumor independent on T cell. 3×10^5 MC38 were inoculated into Rag-1 mice. 25μg of IFN-α-PDL1 was injected into mice on day 9. Two days later, tumor tissues were harvested, and PD-L1 levels were determined by flow cytometry. Data is a representative of two independent experiments.

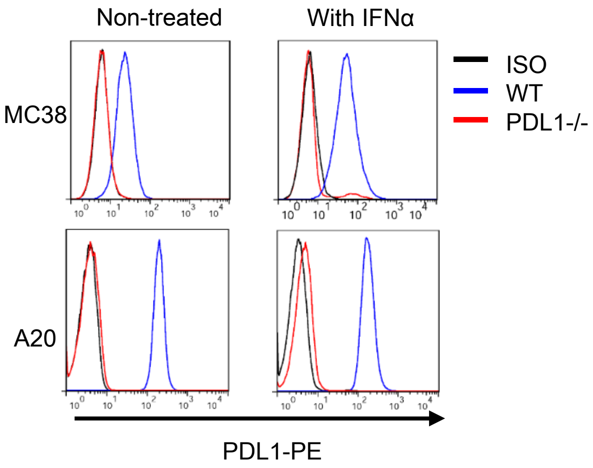

Supplementary Figure 8: Identification of PD-L1 deficient MC38 and A20 cell lines. WT or PD-L1 deficient MC38 or A20 cells were cultured with or without 20 ng/ml IFNα-Fc for 24 hours. PD-L1 expression levels were determined by FACS. Data is a representative of three independent experiments.

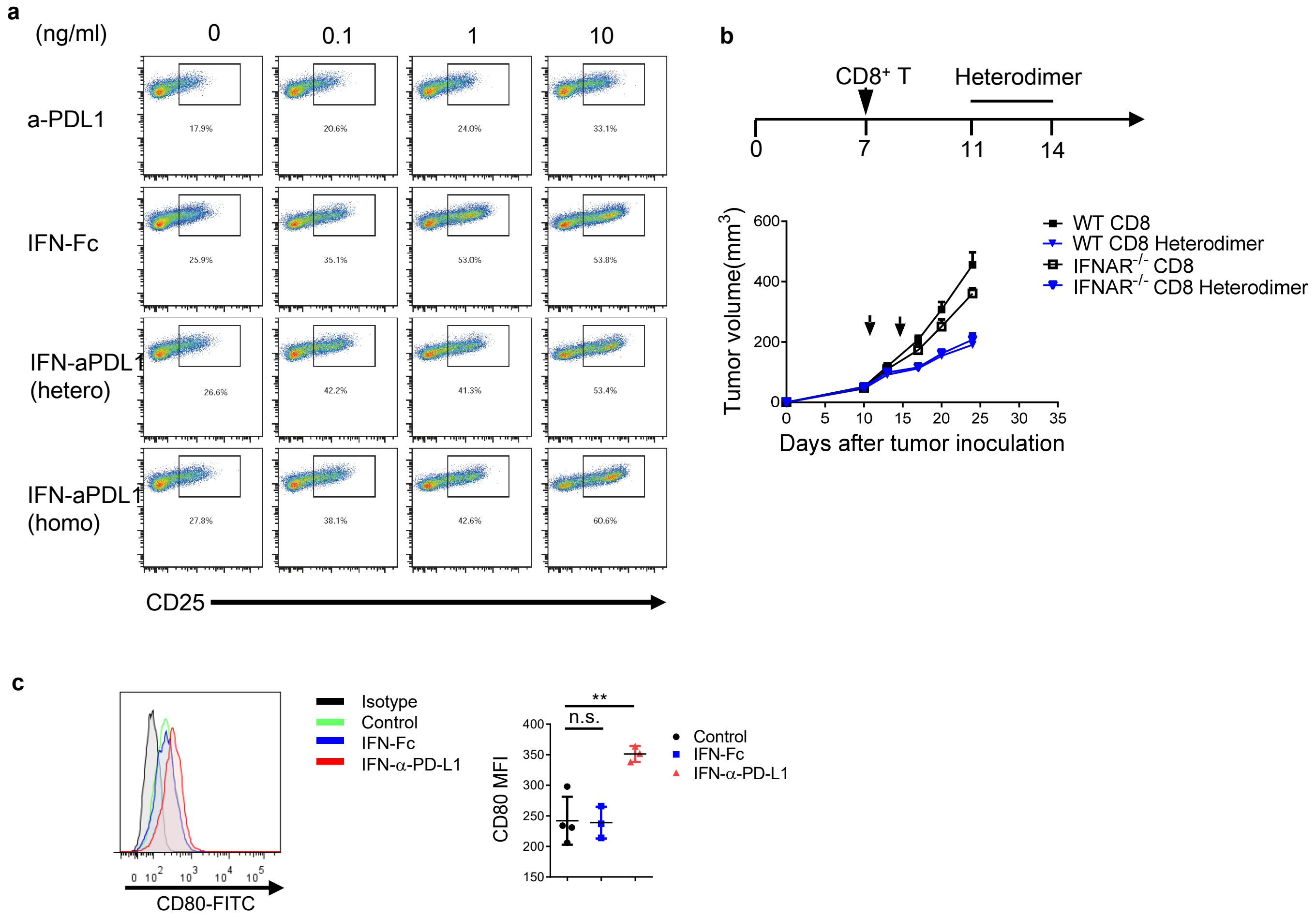

Supplementary Figure 9: IFNAR signaling on CD8 T cells play minor roles in tumor control by IFNα-anti-PDL1. (a) Splenic $CD8^+$ T cells were sorted and allocated into anti-CD3 (1 μg/ml) coated 96-well plate, and cultured in the presence of different concentrations of IFNα-anti-PD-L1 or control proteins for 24 hrs. CD25 expression were measured by FACS. (b) B6.$Rag1^{-/-}$ mice were inoculated with 3×10^5 MC38 cells. 1×10^6 sorted $CD8^+$ T cells from WT or $IFNAR1^{-/-}$ mice were injected intravenously into tumor bearing $Rag1^{-/-}$ mice on day 7. Mice were treated with 25 μg IFNα-anti-PD-L1 on day 11 and 14. Tumor growth was measured twice a week. (c) Two days after IFNα-anti-PD-L1 treatment, MC38 tumor tissues were isolated. The expression of CD80 in tumor-infiltrating DCs ($CD11c^+MHCII^+$) was measured by flow cytometry. Data is a representative of at least two independent experiments.

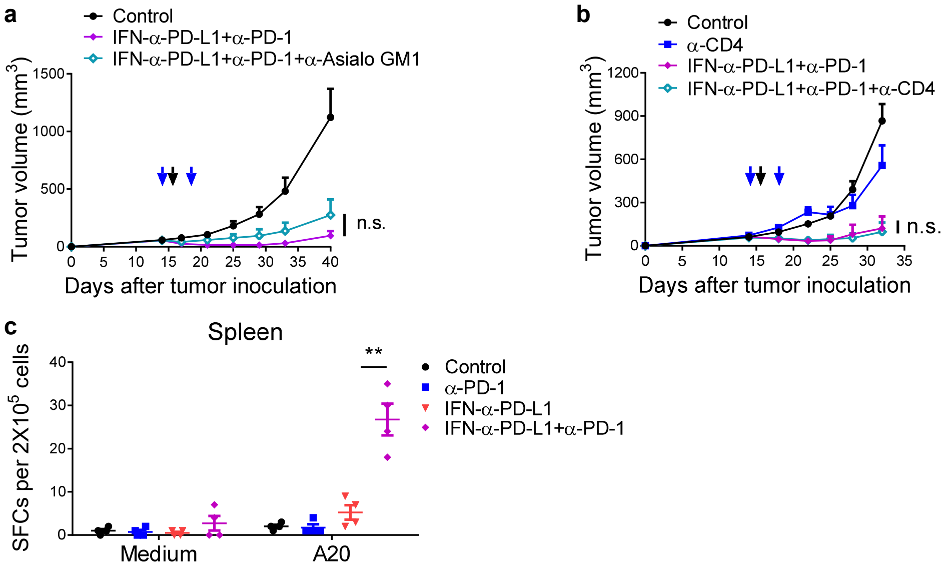

Supplementary Figure 10: NK and CD4^{+} T cells play minor roles in antitumor responses mediated by anti-PD-1 and IFNα-anti-PD-L1. A20 tumor-bearing mice (n=4-5) were treated as in Figure 6A. (a) To deplete NK cells, 20 µg of anti-asialo GM1 was injected i.p. twice weekly from day 13. (b) For CD4^{+} T-cell depletion, 200 µg of anti-CD4 was administered i.p. twice weekly from day 13. Tumor growth was measured twice per week. Data indicates mean ± SEM and is a representative of two experimental replicates. Black and blue arrows indicate treatment with anti-PD-1 and IFNα-anti-PD-L1, respectively. (c) Mice were treated as in Fig. 6e, spleen was isolated, and single-cell suspensions were prepared. Cells were co-cultured with or without irradiated A20. An IFNγ ELISPOT assay was performed. Data indicates mean ± SEM.

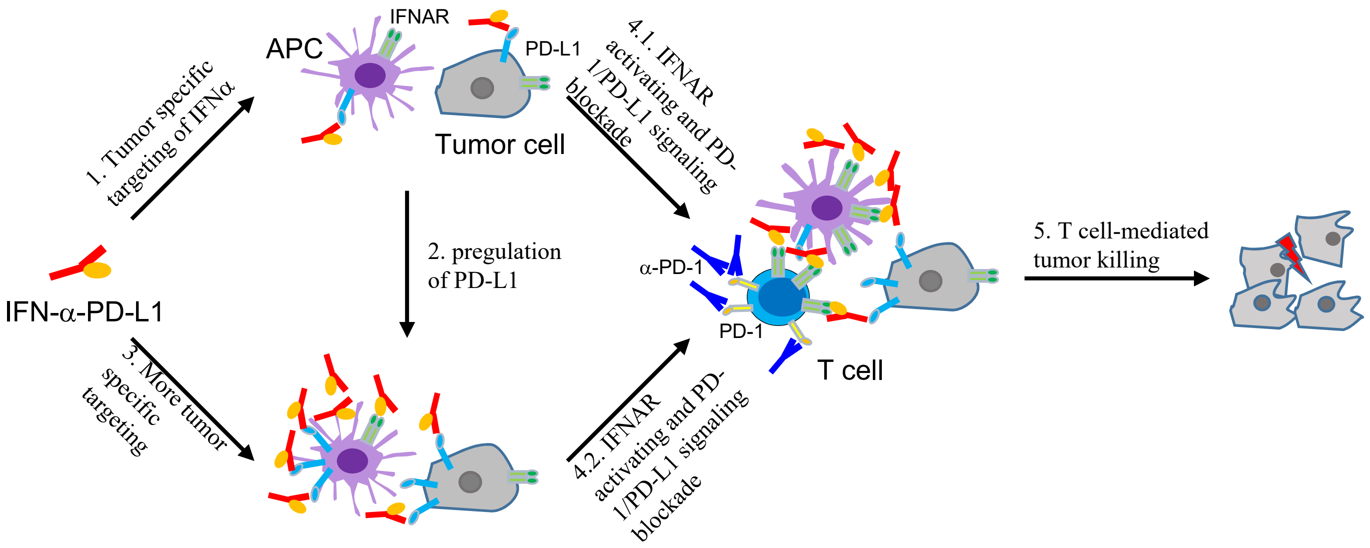

Supplementary Figure 11: Schematic model of antitumor effects mediated by IFNα-armed anti-PD-L1. IFNα-anti-PD-L1 creates multiple feedforward loops to maximize the antitumor effects. Anti-PD-L1 introduces IFNα specifically into tumor tissues (1). IFNα-mediated upregulation of PD-L1 enhances tumor-specific targeting (2 and 3). The antibody blocks PD-L1/PD-1 to rescue PD-L1-driven T-cell exhaustion (4). In addition, IFNα-anti-PD-L1 activating IFNAR signaling in the host to boost T cell activating (4). Together, these factors (re-)activated T-cell responses to control tumor growth (5).
